# Medical physics 3.0 versus 1.0: A case study in digital radiography quality control

**DOI:** 10.1002/acm2.12425

**Published:** 2018-08-17

**Authors:** Diana E. Carver, Charles E. Willis, Paul J. Stauduhar, Thomas K. Nishino, Jered R. Wells, Ehsan Samei

**Affiliations:** ^1^ Department of Imaging Physics University of Texas M. D. Anderson Cancer Center Houston TX USA; ^2^ Clinical Imaging Physics Group Duke University Medical Center Durham NC USA; ^3^ Carl E. Ravin Advanced Imaging Laboratories Duke University Medical Center Durham NC USA; ^4^ Present address: Diana E. Carver, Department of Radiology and Radiological Sciences Vanderbilt University Medical Center Nashville TN USA

**Keywords:** data analytics, detector performance, digital radiography, failure mode analysis, quality assurance, quality control

## Abstract

**Purpose:**

The study illustrates how a renewed approach to medical physics, Medical Physics 3.0 (MP3.0), can identify performance decrement of digital radiography (DR) systems when conventional Medical Physics 1.0 (MP1.0) methods fail.

**Methods:**

MP1.0 tests included traditional annual tests plus the manufacturer's automated Quality Assurance Procedures (QAP) of a DR system before and after a radiologist's image quality (IQ) complaint repeated after service intervention. Further analysis was conducted using nontraditional MP3.0 tests including longitudinal review of QAP results from a 15‐yr database, exposure‐dependent signal‐to‐noise (SNR ^2^), clinical IQ, and correlation with the institutional service database. Clinical images were analyzed in terms of IQ metrics by the Duke University Clinical Imaging Physics Group using previously validated software.

**Results:**

Traditional metrics did not indicate discrepant system performance at any time. QAP reported a decrease in contrast‐to‐noise ratio (CNR) after detector replacement, but remained above the manufacturer's action limit. Clinical images showed increased lung noise (Ln), mediastinum noise (Mn), and subdiaphragm‐lung contrast (SLc), and decreased lung gray level (Lgl) following detector replacement. After detector recalibration, QAP CNR improved, but did not return to previous levels. Lgl and SLc no longer significantly differed from before detector recalibration; however, Ln and Mn remained significantly different. Exposure‐dependent SNR ^2^ documented the detector operating within acceptable limits 9 yr previously but subsequently becoming miscalibrated sometime before four prior annual tests. Service records revealed catastrophic failure of the computer containing the original detector calibration from 11 yr prior. It is likely that the incorrect calibration backup file was uploaded at that time.

**Conclusions:**

MP1.0 tests failed to detect substandard system performance, but MP3.0 methods determined the root cause of the problem. MP3.0 exploits the wealth of data with more sensitive performance indicators. Data analytics are powerful tools whose proper application could facilitate early intervention in degraded system performance.

## 1 INTRODUCTION

### 1.A Traditional quality control by clinical medical physicists: Medical Physics 1.0

In the routine course of clinical practice, the medical physicist performs tests and analyzes data intended to indicate whether imaging systems are producing adequate image quality at acceptable radiation doses. The National Commission on Radiation Protection and Measurements (NCRP) states that all members of an imaging facility are responsible for quality control (QC) activities, with ultimate responsibility residing primarily with the imaging physician in charge. However, the responsibility for technical details remains with the medical physicist.^1^ QC tests are typically performed on an incidental basis during acceptance, commissioning, annual inspections, troubleshooting, or performance verifications after service, and are part of an overall quality assurance program.^2^, ^3^, ^4^ The test procedures and pass/fail criteria may come from federal, state, or local regulations, accrediting or professional organizations, adaptions from the scientific literature, or the equipment manufacturers themselves.^5^, ^6^, ^7^ The QC tests are snapshots of system performance in time, and with rare exceptions, there are no firm requirements to compare performance to historical results, to other systems, or to establish trends. A measurement within acceptable criteria is considered to “pass.” Once the system performance level is established, monitoring of its performance is not required until the next inspection or service event.^5^, ^6^, ^7^ This pattern of QC support is what could be called “Medical Physics 1.0 (MP1.0),” the current standard of practice.^8^, ^9^, ^10^, ^11^ Clinical medical physicists exceed this basic level of service as their time, resources, and individual preferences allow, but this description provides a reasonable minimum expectation for physicist testing. This level of QC support is also consistent with the description of “Level 1 services” defined by the American Association of Physicists in Medicine (AAPM) Diagnostic Work and Workforce Study Subcommittee's Levels of Service model in Report 301.^12^ In this sense, MP1.0 tests are “well‐defined, and there is a relatively high degree of agreement among medical physicists on procedures … to perform them.”^12^

### 1.B A new paradigm for the practice of clinical medical physics: Medical Physics 3.0

Medical Physics 3.0 (MP3.0)^13^ (and its earlier, more narrowly defined moniker of Medical Physics 2.0^8^, ^9^, ^10^, ^11^) is an emerging concept within the AAPM and medical physics profession that is incidentally in step with the American College of Radiology's Imaging 3.0™.^14^ The overall purpose of the MP3.0 initiative is to modernize imaging physics specialties, to reestablish relevance to clinical performance, and to improve efficiency. The MP3.0 initiative^15^ encourages medical physicists in clinical practice to:

be more relevant to the clinical setting, … refresh their competency in statistics and data analytics, … in addition to ‘what’ and ‘how,’ [to] better understand ‘why,’… [and to] include optimization of clinical procedures and retrospective analysis of care data, in addition to equipment assurance and inspection.

The Information Age has afforded medical physicists advanced analytical capabilities using both imaging systems themselves and the computers that they employ to acquire and analyze test data. These capabilities allow for a new level of sophistication, efficiency, and sensitivity in QC of imaging systems that could be associated with MP3.0. MP3.0 is a vision for transitioning to value‐ and evidence‐based medicine and aims to expand clinical physics beyond the traditional insular models of testing that could be regarded as MP1.0. MP3.0 is scientifically informed by findings and methods, clinically relevant to the operational practice, and pragmatic in its meaningful and efficient use of resources. Furthermore, MP3.0 strives for quality consistency in addition to compliance, team‐based clinical operation models, and retrospective evaluation of clinical performance. This type of effort in QC support is consistent with “Level 3 services” as defined in AAPM Report 301.^12^

Whereas MP1.0 analysis considers system performance in temporal isolation, MP3.0 may use sophisticated informatics resources to analyze the temporal system performance characteristics. As the medical physicist collects and analyzes historical QC results and establishes trends, the MP3.0 framework exploits the wealth of data through the use of more sensitive performance indicators. As a result, the interval to detection of substandard performance can be decreased.

Herein, a clinical case is described to illustrate these two different QC paradigms. An image quality complaint from a radiologist called for medical physics attention to this case, and root‐cause analysis was subsequently incorporated into an ongoing institutionally approved retrospective quality improvement project.

### 1.C The image quality complaint: Initial actions upon receipt

**Figure 1:**
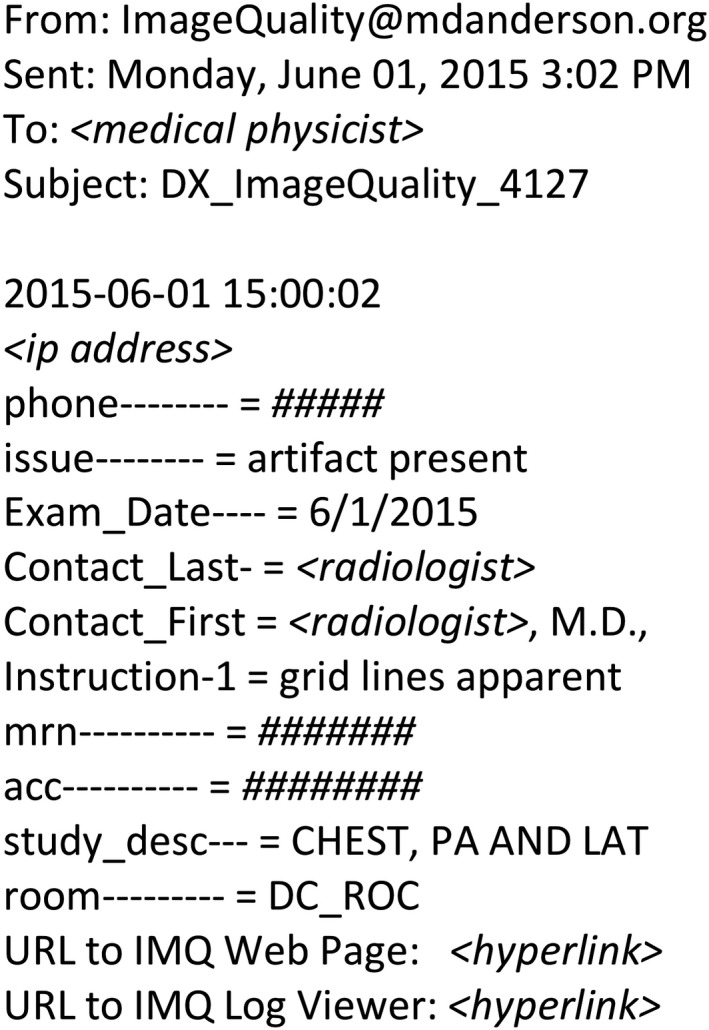
Image quality report documenting radiologist complaint. Application integrated into the PACS viewer sends formatted message via email to predefined distribution list including medical physicist. All fields are automatically populated with the exception of the “issue,” which can be selected from a pull‐down menu or input free‐text, and four available lines of free‐text for further description of the problem. The contact name and phone can be over‐ridden. The reports are archived and used to track action on complaints.

**Figure 2:**
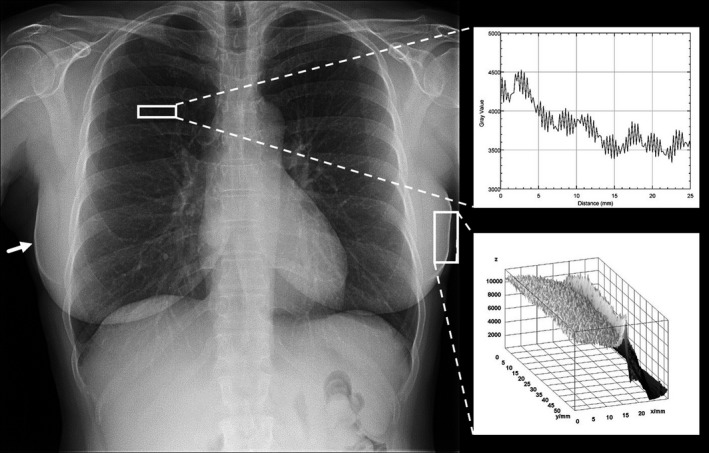
PA chest radiograph that prompted radiologist's image quality complaint on June 1, 2015, for prominent grid lines. Rebound artifacts — also known as “Uberschwinger” artifacts^16^, ^17^, ^18^ — were noted as indicated by the arrow. This is also seen in exaggerated contrast of some cortical bone. Upper right inset: line profile shows exaggerated grid lines in image. The prominent beat frequency corresponds to the aliased frequency of the grid. Lower right inset: surface plot of one of the rebound artifacts.

This study was motivated by a radiologist's image quality complaint on June 1, 2015, regarding a digital radiograph acquired using a commercial digital radiography (DR) unit (Revolution XQi, GE Healthcare, Milwaukee, WI). The complaint was documented through an automated reporting application that MD Anderson had developed and integrated into the PACS system in 2005 (Fig. 1). The specific complaint was that grid lines were very prominent on the posteroanterior (PA) view of the radiograph (Fig. 2). Upon inspection, the exaggerated grid lines were verified; however, white artifacts along the skin lines and cortical bones were also visible on the PA and lateral (LAT) views. These image processing artifacts along regions of rapidly changing density are also known as rebound or “Uberschwinger” artifacts.^16^, ^17^, ^18^ Prior clinical experience with these artifacts suggested that improper detector gain and offset calibration was a likely cause of both this and the prominent grid lines.

A proper detector gain and offset calibration can reduce the appearance of both rebound artifacts and grid lines present in clinical images. In the DR system, the grid is located in a fixed position relative to the detector. At a given source‐to‐image distance (SID), the grid lines are projected onto the detector in the exact same location except for any slight deviation from perfect alignment of the x‐ray tube and the grid/image receptor. The projection of the grid lines imposes a periodic nonuniformity in exposure across the detector. If the gain and offset calibration is performed by means of a flat‐field acquisition with the grid in place, this nonuniformity tends to be corrected.^19^, ^20^ If this calibration is not performed properly, the digital image processing algorithm can aggravate this periodic nonuniformity, which then manifests itself to the radiologist as “prominent grid lines.”^19^, ^20^

**Figure 3:**
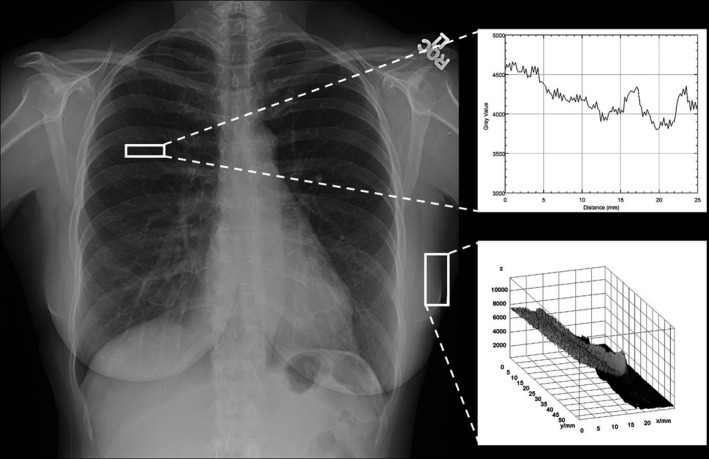
Corrective action was successful. PA chest radiograph of first female patient taken after detector recalibration showed less prominent grid lines and dramatically reduced rebound artifacts. Upper right inset: line profile shows grid lines are less pronounced. Lower right inset: surface plot demonstrates reduced rebound artifact.

A service call was made for recalibration of the detector, and the system was removed from clinical use. The detector was recalibrated for gain and offset, and a manufacturer Quality Assurance Procedures (QAP) test was performed. The DR unit passed the QAP test and was returned to clinical use. Afterward, clinical images obtained using the system no longer exhibited excessive grid lines or rebound artifacts (Fig. 3).

Overall, corrective action was successful, but why did routine QC measurements not warn of the problem sooner? Apparently, routine QC measurements were either not designed or not optimized to detect the cause of these artifacts. Additionally, how long had the system been producing substandard images, and could there be other measurements that might have been more prognosticative? To address these questions, root‐cause analysis was initiated using four advanced methods: inspection of a database containing QAP test results, exploration of clinical image quality metrics, analysis of exposure‐dependent signal‐to‐noise ratio squared (SNR^2^) data, and queries of the institutional service events record database.

## 2 MATERIALS AND METHODS

### 2.A QAP database

**Figure 4:**
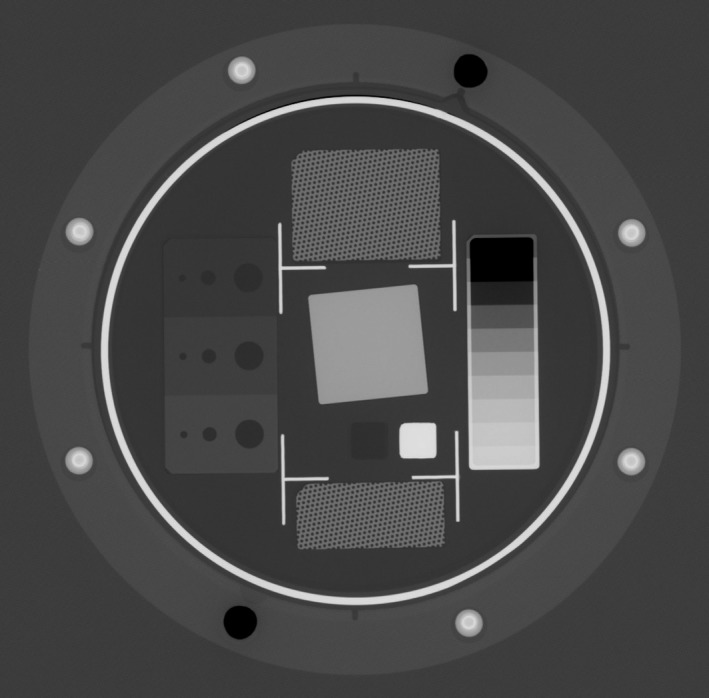
DR image of the GE Healthcare IQST phantom for the Revolution XQi system.

MD Anderson performs QAP testing for all GE DR systems weekly and archives the results in a database dating back to 2001. The QAP test includes exposure and automatic analysis of two uniform images through a 20 mm Al filter and an image of the Image Quality Signature Test (IQST) phantom acquired at 80 kVp, 20 mAs, with fixed 180 cm SID, 13:1, 78 lines/cm, Al interspace grid, with 29 μm Pb septal thickness (Fig. 4).^21^ The two uniform images are analyzed to determine artifacts, local and global brightness nonuniformity, and SNR nonuniformity. The IQST phantom contains inserts for measuring spatial modulation transfer function (MTF), dynamic range linearity and accuracy, resolution nonuniformity, electronic and correlated noise, and contrast‐to‐noise ratio (CNR), which proved to be of particular value in this case.

**Figure 5:**
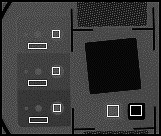
IQST schematic showing ROI placement for CNR calculation. Schematic is inverse grayscale of central portion of Fig. 3. Difference in mean gray level between three pairs of rectangular and square ROIs (contrasts) are each divided by a value for noise derived from the square ROIs to yield CNR1, CNR2, and CNR3. The two square ROIs on the right side of the schematic are used in the calculation of MTF from the edge phantom.

The QAP analysis software automatically calculates CNR^22^ for three different contrast levels in the “for‐processing” (*aka* “raw, ranged,” “unprocessed”) image of the IQST phantom (Fig. 4). The calculations are made using three pairs of rectangular and square regions of interest (ROIs) located on the left side of the central portion of the IQST image (see Fig. 5). The difference in the mean gray level between each rectangle and its corresponding square is defined as the contrast. Each square ROI is also used to calculate the value for noise. CNR is reported as CNR1, CNR2, and CNR3 from low to high contrast level, respectively.^1^

MD Anderson uses a custom software program to retrieve the vendor‐generated QAP test results from each machine, parse the files, and store the results in a database. A website displays the long‐term test results for review. The program was developed in Windows^®^ (Microsoft Corporation, Redmond, WA) utilizing a web development platform called WAMP^®^, an installation package consisting of the Apache^®^ (web server), MySQL^®^ (database), and PHP^®^ (server‐side scripting language) packages distributed on the web by Bitnami (San Francisco, CA). Data are visualized using jqPlot^®^, a JavaScript^®^ plotting, and charting plug‐in. All of the software used in the development of this program is free and open‐source. This software resides on a server in the hospital with file transfer protocol (FTP) access through interposing firewalls to the imaging devices. The program updates the database automatically and on demand by querying each device to determine whether new QAP results are available. Because different GE Healthcare DR models and software versions report different sets of QAP metrics,^21^ the database was designed to accommodate these variations. Beyond the automatic collection of results, the major benefit of the software is its ability to display longitudinal results to uncover trends. The numerical values can be exported from the database.

### 2.B Exposure‐dependent SNR^2^

Exposure‐dependent SNR^2^ measurements, which are analogous to the noise‐equivalent quanta of an image (NEQ), provide criteria for analyzing the performance of digital flat‐panel imaging systems.^23^ Gain and offset calibration of the detector has been shown to reduce the variation in exposure‐dependent SNR^2^ performance among DR systems. Because these measurements are valuable for identifying abnormal detector performance, the next step in the root‐cause analysis was to compare the exposure‐dependent SNR^2^ measurements from the Revolution XQi system with established confidence limits.^23^ MD Anderson routinely acquires SNR^2^ as a function of exposure as part of DR annual testing, so these data were available from annual reports.

### 2.C Clinical image quality metrics

The next step in the root‐cause analysis was to evaluate clinical image quality metrics for individual PA chest radiographs acquired using a Revolution XQi DR unit. Ninety‐three images were chosen for analysis as part of a retrospective review. This evaluation was approved by the MD Anderson institutional Quality Improvement Assurance Board. Based on inspection of the weekly QAP measurements, images were chosen to represent different periods of recorded CNR, consisting of “normal” CNR (17 images), “lower” CNR (20 images), “higher” CNR (20 images), and images acquired during a transitional period from normal to lower CNR (36 images). Upon further examination of the service history, it was apparent that the CNR groups corresponded to service events, i.e., prior to detector replacement, after detector replacement, after detector recalibration, and the 2‐week period immediately after detector replacement, respectively.

**Figure 6:**
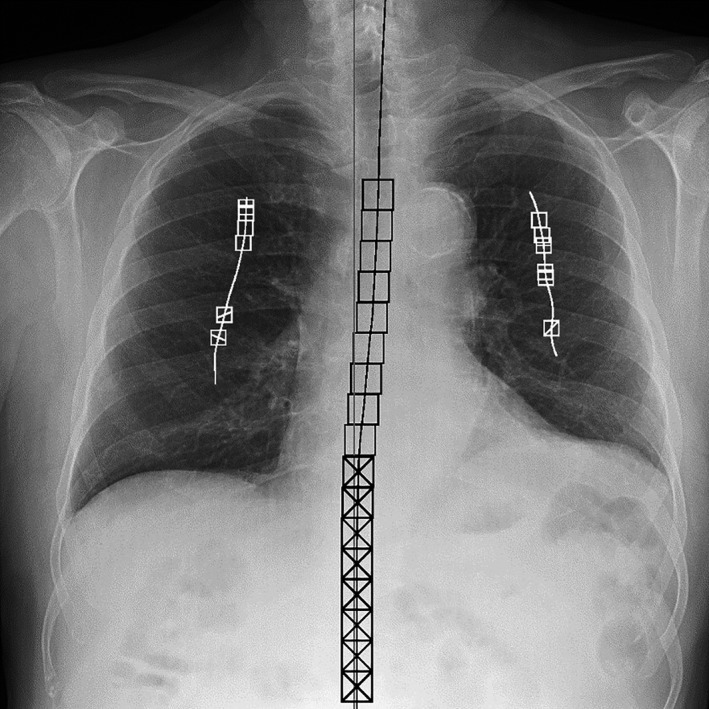
ROI placement example on a PA chest radiograph. White squares without lines are Lung ROIs, while those with a line indicating rib edge crossings are Rib/Lung ROIs. Black squares without crosses are mediastinum ROIs, and those with crossed lines are subdiaphragm ROIs.^24^

The images were anonymized, securely transferred, and analyzed by the Duke University Clinical Imaging Physics Group using a previously described^24^ and validated^25^ software program. The software automatically segments each image and measures 10 perceptual attributes of chest radiographs. The image quality metrics reported for each image consist of lung gray level (Lgl), lung detail (Ld), lung noise (Ln), rib‐lung contrast (RLc), rib sharpness (Rs), mediastinum detail (Md), mediastinum noise (Mn), mediastinum alignment (Ma), subdiaphragm‐lung contrast (SLc), and subdiaphragm area (Sa). The segmentation is also recorded for each image in a jpeg thumbnail. An example is shown in Fig. 6.

Descriptive statistics were calculated for image quality metrics for each group of images using the SPSS software program (version 23; IBM Corporation, Armonk, NY). Image quality metrics were compared across groups using a one‐way analysis of variance (ANOVA). The same software program was used to generate control charts.

### 2.D Institutional service database

MD Anderson's service database was queried to reveal events that may have affected detector performance. This database contains records of service events for all diagnostic imaging modalities and auxiliary equipment at MD Anderson. The database was implemented using a customized commercial software program (EAM, version 10; Infor, New York, NY) and is populated semiautomatically from service calls and service reports. Electronic records in the database date back to 2004. Paper records of events before 2004 are available.

### 2.E Integration of performance metrics

The QAP database provided a means to visually assess each of the seventeen QAP metrics before and after the time period when the artifact was observed and reported. Images were analyzed from the same time period, and the resulting values for the ten clinical image quality metrics for groups of images (predetector replacement, postdetector replacement, postdetector recalibration, and the transition groups) were compared statistically to identify which metrics showed substantial changes that were concurrent with the event. The exposure‐dependent SNR^2^ data were used to broaden the search for a root cause of the detector miscalibration. The service database was the ultimate source of an explanation of the unexpected performance changes.

## 3 RESULTS

### 3.A QAP data

The QAP results immediately before the image quality complaint and immediately after the detector recalibration are shown in Table 1. All of these results passed GE's recommended action limits. The values of the metrics before and after calibration are essentially the same with the exception of a slight decrease in global brightness nonuniformity, which is usually observed after detector gain calibration, and a dramatic increase in all three CNR levels (approximately a factor of two). Review of the historical QAP test results revealed that the CNR values were abnormally low dating back to a time in close proximity to a detector replacement.

**Table 1:** QAP results before and after detector gain and offset calibration. Contrast‐to‐noise ratio values are emphasized to indicate that these were the only values to display large differences before and after detector replacement. The abbreviations, “LSL” and “USL”, stand for “lower system limit” and “upper system limit,” respectively

Test	2015‐05‐29	2015‐06‐01	LSL	USL	Result
Artifacts — number of bad pixels	0.00	0.00	N/A	10.00	PASS
Brightness nonuniformity — global	2.81	1.78	N/A	10.00	PASS
Brightness nonuniformity — local	0.67	0.61	N/A	5.00	PASS
SNR nonuniformity	11.31	12.23	N/A	40.00	PASS
Spatial MTF at 0.5 lp/mm	91.52	91.30	70.00	N/A	PASS
Spatial MTF at 1.0 lp/mm	75.43	75.79	53.00	N/A	PASS
Spatial MTF at 1.5 lp/mm	58.82	58.39	35.00	N/A	PASS
Spatial MTF at 2.0 lp/mm	40.74	40.94	23.00	N/A	PASS
Spatial MTF at 2.5 lp/mm	30.38	28.30	17.00	N/A	PASS
Dynamic range — level linearity	0.97	0.97	N/A	N/A	PASS
Dynamic range — level accuracy	98.71	98.36	90.00	N/A	PASS
***Contrast/noise ratio 1***	***6.85***	***12.91***	***3.00***	***N/A***	***PASS***
***Contrast/noise ratio 2***	***12.27***	***24.50***	***N/A***	***N/A***	***PASS***
***Contrast/noise ratio 3***	***18.53***	***32.17***	***N/A***	***N/A***	***PASS***
Resolution nonuniformity	9.10	6.99	N/A	40.00	PASS
Elec. Noise	4824.00	4831.00	N/A	N/A	PASS
Correlated	8.00	8.00	N/A	N/A	PASS

**Figure 7:**
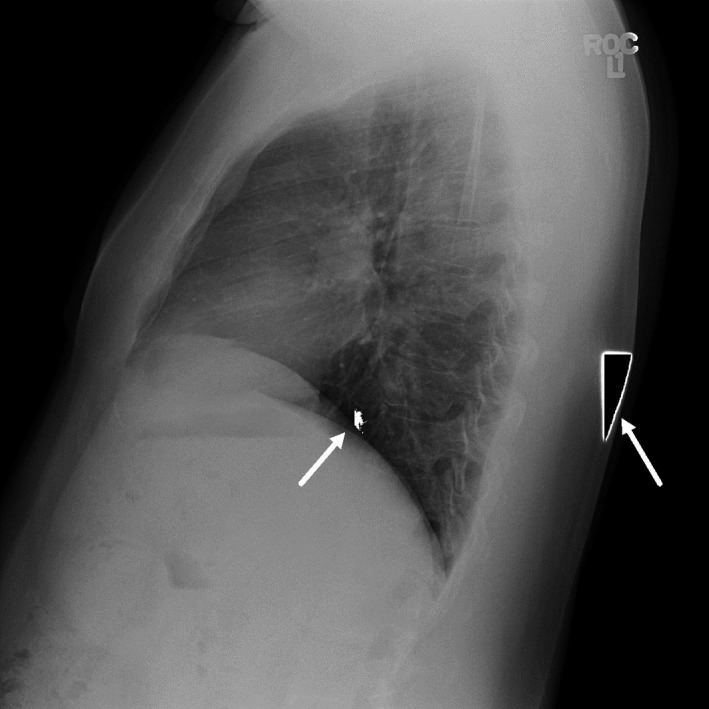
LAT chest radiograph showing artifacts (white arrows) that appeared suddenly, necessitating a detector replacement on August 5, 2014.

**Figure 8:**
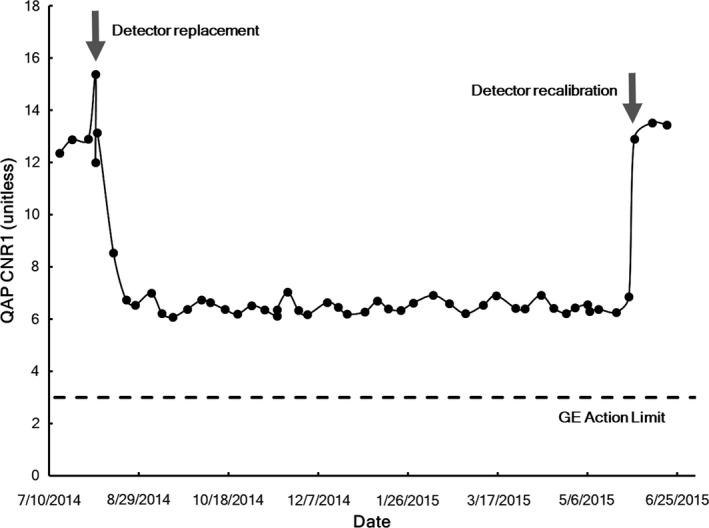
Longitudinal GE Healthcare QAP results. CNR1 values are shown as points, and the minimum acceptable limit set by GE Healthcare is shown. A custom software program retrieves weekly QAP results from the Revolution XQi system, parses the files, and stores the results in a database. Arrows indicate the detector replacement necessitated by the artifact shown in Fig. 6 and the detector recalibration performed after the artifacts noted in Fig. 1.

The detector replacement was necessitated by the sudden appearance of gross artifacts, shown in Fig. 7, which could not be corrected by recalibration. The most recent annual test was performed on August 5, 2014, in conjunction with the detector replacement, and it passed all of the required tests. The QAP test performed immediately afterward indicated a normal CNR level as shown in Fig. 8. However, the following week's QAP test had a lower CNR, and the CNRs from subsequent QAP tests fell to low levels. Recalibration of the system's detector on June 1, 2015, following the image quality complaint, restored the CNR back to moderate levels.

### 3.B Clinical image quality metrics

Descriptive statistics for image quality metrics are reported in Table 2 for six different groups: predetector replacement, postdetector replacement, postdetector recalibration, the entire 2‐week transition period from high to low CNR based on the QAP data, the first week of the transition period, and the second week of the transition period. The mean, standard deviation (SD), and 95% confidence interval (CI) lower and upper limits were calculated for the statistics in each of these groups.

**Table 2:** Descriptive statistics for clinical image quality metrics

	Lgl	Ld	Ln	RLc	Rs	Md	Mn	Ma	SLc	Sa
Pre detector replacement N = 17 07/22/14–08/04/14										
Mean	3837.2	96.0	55.5	0.2	89.5	33.8	77.6	7.3	1.0	0.3
Std. Dev.	50.9	2.9	1.7	0.0	0.0	2.8	2.0	1.3	0.0	0.0
95% lower	3729.2	89.8	51.9	0.2	89.5	27.8	73.4	4.5	1.0	0.3
95% upper	3945.2	102.2	59.1	0.2	89.6	39.7	81.8	10.1	1.0	0.3
Post detector replacement N = 20 08/25/14–05/28/15										
Mean	3577.1	97.5	80.8	0.2	89.5	33.1	109.5	8.1	1.1	0.3
Std. Dev.	58.8	2.3	1.9	0.0	0.0	2.9	1.7	1.1	0.0	0.0
95% lower	3454.5	92.7	76.8	0.2	89.5	27.1	106.0	5.8	1.0	0.3
95% upper	3699.7	102.3	84.7	0.2	89.6	39.1	113.0	10.4	1.1	0.3
Post detector recalibration N = 20 06/02/15–06/18/15										
Mean	3827.0	93.3	61.3	0.2	89.6	39.2	100.1	6.8	1.0	0.3
Std. Dev.	48.3	2.5	1.6	0.0	0.0	3.5	1.3	1.2	0.0	0.0
95% lower	3725.9	88.1	58.0	0.2	89.5	31.9	97.3	4.4	1.0	0.3
95% upper	3928.0	98.5	64.6	0.2	89.6	46.5	102.9	9.3	1.0	0.3
Transition Pool N = 36 08/06/14–08/21/14										
Mean	3680.1	97.2	72.5	0.2	89.5	31.9	105.1	7.4	1.0	0.3
Std. Dev.	34.8	2.0	1.2	0.0	0.0	1.8	0.8	0.9	0.0	0.0
95% lower	3609.4	93.2	70.1	0.2	89.5	28.3	103.4	5.6	1.0	0.3
95% upper	3750.8	101.2	74.8	0.2	89.6	35.5	106.8	9.2	1.1	0.3
Transition 1st week N = 19 08/06/14–08/14/14										
Mean	3699.9	98.1	69.1	0.2	89.5	32.6	104.1	8.4	1.0	0.3
Std. Dev.	48.1	3.2	1.2	0.0	0.0	2.7	1.1	1.3	0.0	0.0
95% lower	3598.8	91.3	66.6	0.2	89.5	27.0	101.8	5.6	1.0	0.3
95% upper	3801.0	104.9	71.7	0.2	89.6	38.2	106.5	11.1	1.1	0.3
Transition 2nd week N = 17 08/15/14–08/21/14										
Mean	3670.3	98.1	76.5	0.2	89.5	30.4	105.9	6.1	1.0	0.3
Std. Dev.	53.3	2.3	1.7	0.0	0.0	2.4	1.3	1.2	0.0	0.0
95% lower	3556.8	91.3	72.9	0.2	89.5	25.3	103.0	3.6	1.0	0.3
95% upper	3783.8	104.9	80.1	0.2	89.6	35.4	108.8	8.7	1.1	0.4

The image quality metrics of five of the groups are compared in Table 2, excluding the pooled weeks of transition from high to low CNR. The results of ANOVA for these paired comparisons are shown in Table 3. No statistically significant differences were observed between groups in lung detail (Ld), rib‐lung contrast (RLc), rib sharpness (Rs), mediastinum detail (Md), mediastinum alignment (Ma), or subdiaphragm area (Sa).

**Table 3:** ANOVA results for comparisons of image quality metrics. Bolded values indicate statistically significant differences (*P* < 0.05)

Comparison		Lgl	Ld	Ln	RLc	Rs	Md	Mn	Ma	SLc	Sa
I	Prereplacement vs Postreplacement	**0.002**	0.679	**0.000**	0.137	0.513	0.871	**0.000**	0.670	**0.012**	0.575
II	Prereplacement vs Postrecalibration	0.885	0.482	**0.017**	0.588	0.400	0.244	**0.000**	0.770	0.714	0.089
III	Postreplacement vs Postrecalibration	**0.002**	0.216	**0.000**	0.066	0.832	0.183	**0.000**	0.440	**0.002**	0.288
IV	Prereplacement vs Transition week 1	0.058	0.633	**0.000**	0.683	0.790	0.766	**0.000**	0.581	0.204	0.243
V	Transition week 1 vs Transition week 2	0.683	0.703	**0.001**	0.830	0.963	0.542	0.317	0.224	0.947	0.878
VI	Postreplacement vs Transition week 2	0.263	0.766	0.115	0.484	0.664	0.488	0.120	0.249	0.135	0.438

ANOVA did reveal statistically significant differences (*P* < 0.05; indicated in bold print) in Lgl, Ln, Mn, and SLc between the predetector replacement and postdetector replacement groups (Comparison I) and between the postdetector replacement and postdetector recalibration groups (Comparison III). However, ANOVA did not suggest any difference in Lgl or SLc between the predetector replacement and postdetector recalibration groups (Comparison II), or during the transitional decrease in CNR (Comparisons IV, V, and VI). Ln and Mn were statistically significantly different from before the detector replacement and after the detector was recalibrated (Comparison II) through transition week 1 (Comparison IV). Ln was the only metric that remained statistically significantly different through transition week 2 (Comparison V). Statistically significant differences were not observed for any metric between postdetector replacement and transition week 2 (Comparison VI). This analysis revealed that the change in performance had occurred by week two of the transition period, and all of the images in week two could have been pooled with the images in the postreplacement group.

The ANOVA results indicated that of all the metrics, Ln was the most correlated with changes in QAP reported CNR. Specifically, Ln was the only image quality metric to change significantly between transition weeks 1 and 2 during the decrease in QAP CNR. Ln changed the most within the first week of the transition, while the QAP test reached only one‐half of its ultimate decrease in CNR over the same interval.

**Figure 9:**
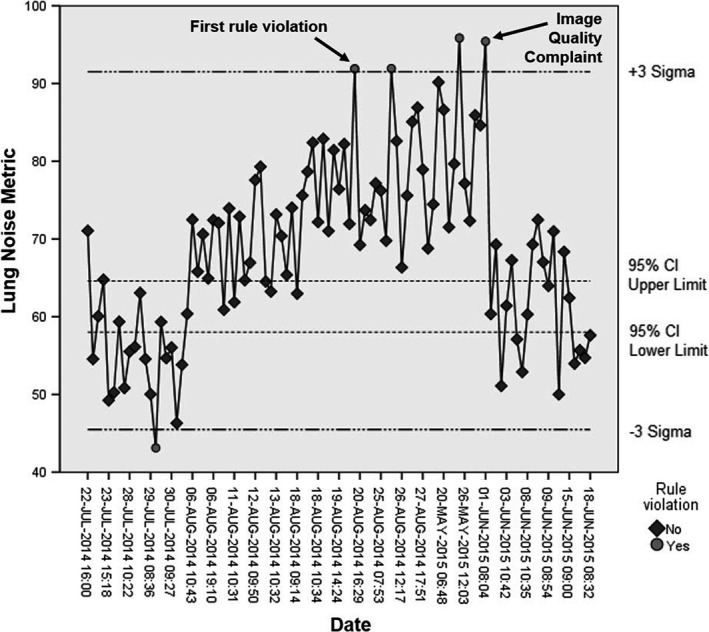
Control chart of the Ln image quality metric. The 95% CI was determined using postrecalibration data. The three‐sigma levels were based on the entire set of data. First rule violation (open circles) for excessive Ln occurred on August 20, 2014, prior to the radiologist complaint on June 1, 2015. Note: events on horizontal axis not equally spaced in time.

A control chart for Ln with limits established from the results of the descriptive statistics for image quality metrics is shown in Fig. 9. The lower and upper limits for the 95% CI for Ln are those for the postdetector recalibration group, and the three‐sigma levels are those taken from the entire image data set. Had action limits been previously established, these would have provided earlier warnings of performance issues with the Revolution XQi system. Specifically, the first rule violation on August 20, 2014 would have signaled abnormal detector performance and provided notification of a detector miscalibration well in advance of the radiologist's image quality complaint on June 1, 2015.

### 3.C Exposure‐dependent SNR^2^

**Figure 10:**
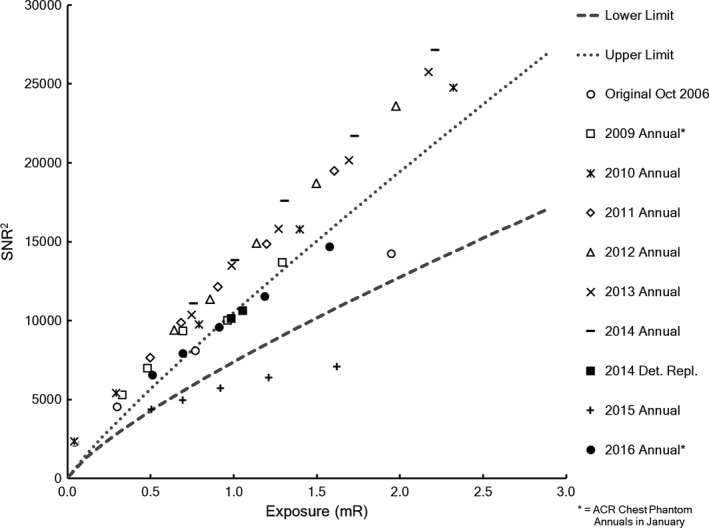
Exposure‐dependent SNR ^2^ measurements for the DR system. Comparison of measurements from annual testing with established limits (broken lines) indicated that the detector was improperly calibrated sometime from 2006 to 2009 but that proper calibration was restored in 2016. The 2014 detector replacement data were collected after the 2014 annual data and the 2015 annual data are contemporary to this case study. Data from the annual tests in 2007 and 2008 were not included because the MD Anderson DR annual testing protocol was not well established at that time.

The initial set of exposure‐dependent SNR^2^ measurements was made in October 2006 soon after acceptance testing of the unit and calibration of the new detector. Unfortunately, similar measurements were not acquired during annual performance evaluation in 2007 and 2008. Exposure‐dependent SNR^2^ measurements were acquired during annual performance evaluation each year from 2009 to 2016, but the results were not analyzed until this retrospective study. The analysis revealed that the detector was operating within acceptable limits in October 2006, but an event occurred before the 2009 data were acquired that caused improper calibration of the detector as shown by abnormally *high* SNR^2^ values. All subsequent data acquired during annual testing through 2014 demonstrated similar improper calibration of the detector (Fig. 10). The exposure‐dependent SNR^2^ measurements also confirmed improper detector calibration after detector replacement, which was reflected in the 2015 annual performance evaluation.

The 2015 annual test was performed in January, in the middle of the period between the detector replacement and the detector recalibration (see Fig. 8). This test was unique in that it indicated performance well *below* the lower acceptable limit. Clinical images acquired during the same time showed rebound artifacts and prominent grid lines similar to the ones shown in Fig. 2 that prompted the radiologist complaint. In fact, the rebound artifacts and prominent gridlines appeared in clinical images on Aug 11, 2014 briefly after the detector replacement and acceptance testing. The artifacts were present in clinical images throughout the 10‐month period until the radiologist's complaint.

### 3.D Institutional service database

**Figure 11:**
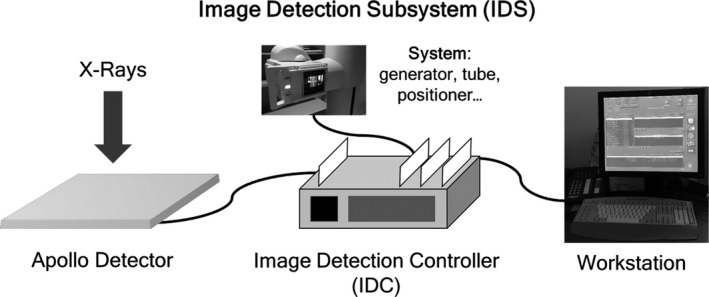
First‐generation GE Healthcare DR systems consist of a constellation of computers, including an IDC.

To discover an event that caused the improper detector calibration, the institutional service events records database for this DR system was the next logical source of information. Queries of the institutional service database revealed several relevant service events between acquisition of the original exposure‐dependent SNR^2^ data in October 2006 and the annual performance evaluation in 2009. These events included a catastrophic failure of the Image Detection Controller (IDC) that contained the original detector calibration data in June 2007. First‐generation GE Healthcare DR systems, including the Revolution XQi, are distributed systems, that is multiple computers connected on a network (Fig. 11), including the IDC. The IDC computer stores the gain and offset calibration, bad pixel map, and conversion factor calibration files.

**Figure 12:**
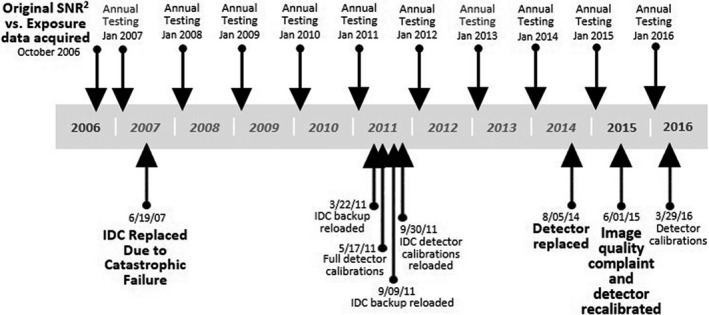
Timeline of QC testing and service events. The first annual test that included measurement of SNR ^2^ vs exposure with use of the appropriate phantom for comparison was in January 2010. If these data had been compared with limits for exposure‐dependent SNR ^2^ (Fig. 9), the problem with the calibration file would have been indicated earlier.

After this catastrophic failure, the IDC was replaced, but according to service records, a new detector calibration was not performed and saved to the new IDC. Instead, an old backup file from prior to October 2006 was loaded onto the new IDC. Furthermore, in subsequent efforts to calibrate the detector, files could not be saved to the new IDC. This continued until the detector failure and replacement in August 2014, in which the calibration file was completely inappropriate for the new detector. Unfortunately, although the new detector appeared to be calibrated properly at acceptance, the calibration file was not properly saved as demonstrated by the 2015 annual testing data (Fig. 10). The timeline in Fig. 12 summarizes QC test results and service events for this DR unit.

## 4 DISCUSSION

The longitudinal QAP database has more utility than the limited application in this case. Inspecting the QAP test results retrospectively allowed identification of a problem with an imaging system: a sudden decrease in CNR following a detector replacement. If trends in the QAP data had been monitored on an ongoing basis, the change would have been noticed much sooner than the radiologist complaint. However, any corrective action would have also required a more stringent CNR action threshold than that which was established by the vendor. Modifying the vendor's action threshold based on analysis of longitudinal performance results is characteristic of MP3.0. This case has reinforced the importance of ongoing monitoring of the QAP database, especially after service events. Now, newly acquired QAP results are compared with past trends to judge whether the results reflect consistent system performance. This approach is limited, however, because the QAP test is only performed weekly. Experience has shown some variation in QAP values depending on actions by the operator. Also, whether the vendor's default action limits are appropriate or simply convenient is uncertain. From studying MD Anderson's wealth of data from multiple DR systems, it should be possible to establish better action limits.

The clinical image quality metrics for the chest radiographs in this case provided a new level of sophistication for root‐cause analysis. Ten perceptual attributes of patient image quality were calculated on an image‐by‐image basis, and the Ln metric was found to be closely correlated with changes in detector performance. A control chart was created for Ln demonstrating that, had Ln been monitored in the images, it would have warned of abnormal performance far ahead of the radiologist's complaint. Because the radiologists continued to interpret suboptimal images with exaggerated grid lines and skin line artifacts for 10 months before reporting them, the data suggest that the automated software is more sensitive to changes in system performance than are human observers — even highly trained radiologists. This is consistent with other findings using these clinical image quality metrics.^26^

Exposure‐dependent SNR^2^ data represent an interesting example of a MP3.0 method sitting idle in its place of creation. Although the method and limits were published in 2011, they were based on data collected in 2006. The protocol was incorporated into every annual performance evaluation starting in 2010, although the data were not compared with limits. The corrective action for a detector showing SNR^2^ performance outside of limits is gain and offset calibration, which is exactly the same corrective action needed for the rebound artifact and pronounced grid lines. Had the data been compared with limits, the problem would have been proactively corrected 5 years before the radiologist's complaint. These data can help spot abnormal detector performance like an improper gain and offset calibration in this case. They could also help identify a defective detector or even a defective grid. Because the limits have been published, any clinical medical physicist can implement this method along with their other annual test procedures on similar radiographic units.

**Figure 13:**
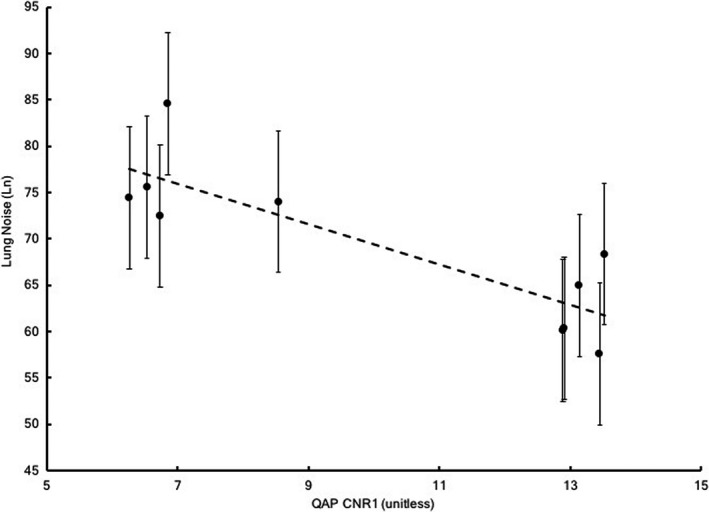
Lung Noise (Ln) vs QAP CNR1. Ln was taken from clinical images acquired within one day of QAP tests. Error bars signify one standard deviation in Ln calculated from the entire clinical dataset for this study. A Pearson correlation coefficient of −0.85, indicating a high negative correlation, was calculated from simple linear regression.

Although QAP CNR and Ln are independent metrics derived from totally different distinct radiographic images, subjects, and features, they both provided an indication of abnormal detector performance and confirmation of corrected performance. This observation leads to the question of whether these two metrics are correlated. To investigate a possible relationship, images that had been analyzed previously were selected if they had been acquired within 2 days of a weekly QAP test. The Ln data from these images were paired with the CNR1 results from the corresponding QAP tests. A plot of Ln vs CNR1 is shown in Fig. 13. A simple linear regression revealed a Pearson correlation coefficient of −0.85, indicating a high negative correlation between these two metrics. It is important to recognize that the Ln values depend on anatomic features and inherently contain quite a bit of variation from patient‐to‐patient, as evidenced by the error bars in Fig. 13. The variation in CNR1 under stable conditions has not been established, however, data in Fig. 8 suggest that ±10% may be a reasonable estimate.

It is interesting to note that while each of the MP3.0 metrics was independently capable of indicating the abnormal detector calibration and the restoration of proper calibration, each has a different fundamental analytical basis. A comparison of the three metrics including the definition, radiographic subject, source image type, and interpretation of each is shown in Table 4. The independent measurements are unique manifestations of the same flawed detector performance.

Ultimately, MD Anderson's institutional service database was key to determining the root cause of the problem in this case. From past experience, the need for detector recalibration was immediately recognized based on the visual appearance of the rebound artifact as soon as the radiologist complained about the image quality. Recalibration immediately corrected the problem. However, without the institutional service database, the root cause of the problem would never have been ascertained, nor would the knowledge have been gained of how to prevent a recurrence of it. In effect, the symptom would have been treated but not the disease. The logical next step is to review the service records further to determine whether past rebound artifact events are also correlated with decreases in QAP CNR.

The results demonstrate that an MP3.0 approach can reveal system performance issues when MP1.0 fails to do so. QAP QC (MP1.0) was consistently performed on time and passed action limits established by the vendor. This weekly test of the system provided only a snapshot of its performance at a moment in time. Retrospective analysis of QAP test results (MP3.0) proved to be better at demonstrating abnormal system performance, because discontinuities in parameter values became readily apparent. Trends in data can easily be determined, and data can be used to predict when action limits will be exceeded, so that detector replacements and other service events can be anticipated. Intercomparisons among systems can be easily accomplished to identify underperforming systems. Visual inspection of each image acquired (MP1.0), either in the technologist's review or, subsequently, by the radiologist, could have indicated the problem in this case sooner. However, relying on human observers elicited an image quality complaint only after months of degraded performance of the system even when an easy means of reporting was readily available. Alternatively, clinical image quality metrics (MP3.0) appeared to be more sensitive performance indicators, and these metrics could be reported with every acquisition rather than at arbitrary (e.g., weekly) intervals. Customization of CIs for individual systems or detectors may be required (statistics and data analytics: a hallmark of MP3.0). The Exposure Index (EI) is another indicator of detector performance and is generally verified at acceptance and during annual testing (MP1.0). EI is a relatively new development that is not available on the DR system in this case. It is unlikely that EI alone would have indicated miscalibration because it reflects only the magnitude of signal rather than the relationship between signal and noise. None of the traditional annual performance evaluations (MP1.0) indicated that the system in this case was performing outside of normal limits. On the other hand, the exposure‐dependent SNR^2^ data (MP3.0) indicated that the detector was miscalibrated. Exploiting the service database is another MP3.0 concept that proved worthwhile in finding an underlying explanation for degraded system performance in this case (part of the “why?”). A comparison of the MP1.0 and MP3.0 methods is shown in Table 5.

**Table 4:** Comparison of MP3.0 metrics

Metric	Definition	Radiographic subject	Source image DICOM presentation intent type	Interpretation
Longitudinal CNR1	Signal difference divided by noise	IQST phantom fixed technique	For‐processing	Contrast resolution of detector for standard exposure
SNR^2^/mR	NEQ surrogate	Patient‐equivalent Phantom AEC	For‐processing	Inherent efficiency of system; calibration of digitization
Lung noise (Ln)	SD of high frequency subband in Lung ROI	Human Patient PA Chest AEC	For‐presentation	Conspicuity of image noise in lung field

The medical physicist, given suitable time and resources, can incorporate MP3.0 methods into their current clinical operations as part of an ongoing, comprehensive QC program. While MP3.0‐inspired methods are promising, their use requires standardization and pragmatic implementation. Meanwhile, MP1.0 methods still offer a pragmatic assurance of equipment quality and thus should not be abandoned prematurely. In some instances, MP1.0 tests provide the foundation upon which the more sophisticated analyses are based. The approaches presented herein were retrospectively applied as part of the root‐cause analysis of the present case, but there are no compelling technical reasons to preclude these methods from being used contemporaneously. Each of these methods has the potential of detecting problems before they impact the clinical imaging operation and in advance of a radiologist's image quality complaint. However, development and fielding of these methods requires an investment of time and resources that must be based on confidence in future benefit of the kind that these results demonstrate.

**Table 5:** MP1.0 vs MP3.0

Test	MP1.0	MP3.0	Frequency of test	Abnormal Performance Detected?
QAP (single instance)	✓		Weekly	No
Longitudinal QAP		✓	Continuous	Yes
Visual Inspection of Images	✓		Twice per acquisition (technologist/radiologist)	No (until image quality report)
Clinical Image Quality Metrics		✓	Once per acquisition	Yes
Exposure Index	✓		Annual and acceptance	No
SNR^2^ vs Detector Exposure		✓	Annual and acceptance	Yes

## 5 CONCLUSIONS

In this case, MP1.0 tests failed to detect substandard DR system performance. All of the traditional tests passed indicating that the system was behaving normally. However, when MP3.0 methods were employed, a problem with the system was not only identified, but also its root cause was determined. This investigation also suggests that the clinical image quality metrics are more sensitive to changes in detector performance than are human observers, as the radiologist's image quality complaint was received nearly a year after the problem originated. A total of 421 patient chest exams were performed on the unit while the problem went undetected. This case demonstrates the necessity of MP3.0. Had these methods been used from the very beginning, awareness of the problem would have occurred much sooner, leading to intervention before it was even noticed in the clinic. Although this case involved the use of a DR system, the principles should extend to other imaging modalities.

## CONFLICTS OF INTEREST

The authors have no relevant conflicts of interest to disclose.

## NOTE

1 Contrast is usually considered to be the difference between the signal behind a feature and its surrounding background. The noise can also be calculated from the background.^22^ In the QAP, each square ROI is located on a hole and each rectangular ROI is located on the plate that achieves the desired signal difference by attenuation, so that the “background” is actually the hole in this case.
